# Supplementary material for: A Molecular Phylogenetic Study of the Genus Phedimus for Tracing the Origin of “Tottori Fujita” Cultivars
Source: Plants (Basel). 2020 Feb 17;9(2):254. doi: 10.3390/plants9020254 (PMC7076367; doi:10.3390/plants9020254)
Supplement: Supplementary file 1 [file plants-09-00254-s001.pdf]

Species	Sample No.	115–118	119–125	126–130	…	265–275
P. takesimensis	K1	ACCA	CGAAAAT	TGGTT		CATTTCAATTT
P. takesimensis	K2	ACCA	CGAAAAT	TGGTT		CATTTCAATTT
P. takesimensis	K3	ACCA	CGAAAAT	TGGTT		CATTTCAATTT
P. takesimensis	K4	ACCA	CGAAAAT	TGGTT		CATTTCAATTT
P. takesimensis	K5	ACCA	ATTTTCG	TGGTT		CATTTCAATTT
P. takesimensis	K6	ACCA	CGAAAAT	TGGTT		CATTTCAATTT
P. takesimensis	K7	ACCA	CGAAAAT	TGGTT		CATTTCAATTT
P. takesimensis	K8	ACCA	CGAAAAT	TGGTT		CATTTCAATTT
P. takesimensis	K9	ACCA	CGAAAAT	TGGTT		CATTTCAATTT
P. takesimensis	K10	ACCA	CGAAAAT	TGGTT		CATTTCAATTT
P. takesimensis	K11	ACCA	ATTTTCG	TGGTT		CATTTCAATTT
P. takesimensis	K12	ACCA	CGAAAAT	TGGTT		CATTTCAATTT
P. takesimensis	K13	ACCA	CGAAAAT	TGGTT		CATTTCAATTT
P. takesimensis	K14	ACCA	CGAAAAT	TGGTT		CATTTCAATTT
P. takesimensis	K15	ACCA	CGAAAAT	TGGTT		CATTTCAATTT
P. takesimensis	K16	ACCA	CGAAAAT	TGGTT		CATTTCAATTT
P. takesimensis	K17	ACCA	ATTTTCG	TGGTT		CATTTCAATTT
P. takesimensis	K18	ACCA	ATTTTCG	TGGTT		CATTTCAATTT
P. takesimensis	K19	ACCA	ATTTTCG	TGGTT		CATTTCAATTT
P. takesimensis	K20	ACCA	CGAAAAT	TGGTT		CATTTCAATTT
P. takesimensis	K21	ACCA	CGAAAAT	TGGTT		CATTTCAATTT
P. takesimensis	K22	ACCA	CGAAAAT	TGGTT		CATTTCAATTT
P. takesimensis	K23	ACCA	CGAAAAT	TGGTT		CATTTCAATTT
P. takesimensis	K24	ACCA	CGAAAAT	TGGTT		CATTTCAATTT
P. takesimensis	K25	ACCA	CGAAAAT	TGGTT		CATTTCAATTT
P. takesimensis	K26	ACCA	CGAAAAT	TGGTT		CATTTCAATTT
P. takesimensis	K27	ACCA	CGAAAAT	TGGTT		CATTTCAATTT
P. takesimensis	J1	ACCA	CGAAAAT	TGGTT		CATTTCAATTT
P. takesimensis	J2	ACCA	CGAAAAT	TGGTT		CATTTCAATTT
P. takesimensis	J3	ACCA	CGAAAAT	TGGTT		CATTTCAATTT
P. takesimensis	J4	ACCA	CGAAAAT	TGGTT		CATTTCAATTT
P. takesimensis	J5	ACCA	CGAAAAT	TGGTT		CATTTCAATTT
P. takesimensis	J6	ACCA	ATTTTCG	TGGTT		CGTTTTAATTT
P. takesimensis	J7	ACCA	ATTTTCG	TGGTT		CGTTTTAATTT
Tottori Fujita 1gou	K55	ACCA	CGAAAAT	TGGTT		CATTTCAATTT
Tottori Fujita 1gou	K56	ACCA	CGAAAAT	TGGTT		CATTTCAATTT
Tottori Fujita 1gou	K57	ACCA	CGAAAAT	TGGTT		CATTTCAATTT
Tottori Fujita 1gou	K58	ACCA	CGAAAAT	TGGTT		CATTTCAATTT
Tottori Fujita 1gou	K59	ACCA	CGAAAAT	TGGTT		CATTTCAATTT
Tottori Fujita 1gou	K60	ACCA	CGAAAAT	TGGTT		CATTTCAATTT
Tottori Fujita 1gou	J15	ACCA	CGAAAAT	TGGTT		CATTTCAATTT
Tottori Fujita 2gou	K61	ACCA	CGAAAAT	TGGTT		CATTTCAATTT
Tottori Fujita 2gou	K62	ACCA	CGAAAAT	TGGTT		CATTTCAATTT
Tottori Fujita 2gou	K63	ACCA	CGAAAAT	TGGTT		CATTTCAATTT
Tottori Fujita 2gou	K64	ACCA	CGAAAAT	TGGTT		CATTTCAATTT
Tottori Fujita 2gou	K65	ACCA	CGAAAAT	TGGTT		CATTTCAATTT
Tottori Fujita 2gou	K66	ACCA	CGAAAAT	TGGTT		CATTTCAATTT
Tottori Fujita 2gou	J16	ACCA	CGAAAAT	TGGTT		CATTTCAATTT
Phedimus sp.	K67	ACCA	CGAAAAT	TGGTT		CATTTCAATTT
Phedimus sp.	K68	ACCA	ATTTTCG	TGGTT		CATTTCAATTT
Phedimus sp.	K69	ACCA	CGAAAAT	TGGTT		CATTTCAATTT
P. kamtschaticus	K28	ACCA	CGAAAAT	TGGTT		CATTTTAATTT
P. kamtschaticus	K29	ACCA	CGAAAAT	TGGTT		CATTTTAATTT
P. kamtschaticus	K30	ACCA	CGAAAAT	TGGTT		CATTTTAATTT
P. kamtschaticus	K31	ACCA	CGAAAAT	TGGTT		CATTTTAATTT
P. kamtschaticus	K32	ACCA	CGAAAAT	TGGTT		CATTTTAATTT
P. kamtschaticus	K33	ACCA	CGAAAAT	TGGTT		CATTTTAATTT
P. kamtschaticus	K34	ACCA	CGAAAAT	TGGTT		CATTTTAATTT
P. kamtschaticus	K35	ACCA	CGAAAAT	TGGTT		CATTTTAATTT
P. kamtschaticus	K36	ACCA	CGAAAAT	TGGTT		CATTTTAATTT
P. kamtschaticus	K37	ACCA	CGAAAAT	TGGTT		CATTTTAATTT
P. kamtschaticus	K38	ACCA	CGAAAAT	TGGTT		CATTTTAATTT
P. kamtschaticus	K39	ACCA	ATTTTCG	TGGTT		CATTTTAATTT
P. kamtschaticus	K40	ACCA	ATTTTCG	TGGTT		CATTTTAATTT
P. kamtschaticus	J8	ACCA	ATTTTCG	TGGTT		CGTTTTAATTT
P. kamtschaticus	J9	ACCA	ATTTTCG	TGGTT		CGTTTTAATTT
P. kamtschaticus	K41	ACCA	ATTTTCG	TGGTT		CGTTTTAATTT
P. aizoon	K42	ACCA	CGAAAAT	TGGTT		CATTTTAATTT
P. aizoon	K43	ACCA	CGAAAAT	TGGTT		CATTTTAATTT
P. aizoon	K44	ACCA	ATTTTCG	TGGTT		CGTTTAAATTT
P. aizoon	K45	ACCA	CGAAAAT	TGGTT		CATTTTAATTT
P. aizoon	K46	ACCA	CGAAAAT	TGGTT		CATTTTAATTT
P. aizoon	K47	ACCA	CGAAAAT	TGGTT		CATTTTAATTT
P. aizoon	J10	ACCA	CGAAAAT	TGGTT		CATTTTAATTT
P. aizoon var. *floribundus*	K48	ACCA	ATTTTCG	TGGTT		CATTTTAATTT
P. aizoon var. *floribundus*	J11	ACCA	CGAAAAT	TGGTT		CATTTTAATTT
P. aizoon var. *floribundus*	J12	ACCA	CGAAAAT	TGGTT		CATTTTAATTT
P. aizoon var. *floribundus*	J13	ACCA	CGAAAAT	TGGTT		CATTTTAATTT
P. aizoon var. *floribundus*	J14	ACCA	CGAAAAT	TGGTT		CATTTTAATTT
P. middendorffianus	K49	ACCA	ATTTTCG	TGGTT		CATTTTAATTT
P. middendorffianus	K50	ACCA	ATTTTCG	TGGTT		CATTTTAATTT
P. middendorffianus	K51	ACCA	ATTTTCG	TGGTT		CATTTTAATTT
P. middendorffianus	K52	ACCA	ATTTTCG	TGGTT		CATTTTAATTT
P. middendorffianus	K53	ACCA	CGAAAAT	TGGTT		CATTTTAATTT
P. middendorffianus	K54	ACCA	ATTTTCG	TGGTT		CATTTTAATTT

Supplementary Figure S1. Three types based on nucleotide substitution in *psb*A-*trn*H sequences of *Phedimus* investigated in the present study including cultivar “Tottori Fujita 1gou” and “Tottori Fujita 2gou”.
